# Two distinct classes of thymic tumors in patients with MEN1 show LOH at the *MEN1* locus

**DOI:** 10.1530/ERC-21-0226

**Published:** 2021-09-10

**Authors:** Adel Mandl, James M Welch, Gayathri Kapoor, Vaishali I Parekh, David S Schrump, R Taylor Ripley, Mary F Walter, Jaydira Del Rivero, Smita Jha, William F Simonds, Robert T Jensen, Lee S Weinstein, Jenny E Blau, Sunita K Agarwal

**Affiliations:** 1 Metabolic Diseases Branch, Metabolic Diseases Branch, National Institute of Diabetes and Digestive and Kidney Diseases, NIH, Bethesda, Maryland, USA; 2 Center for Cancer Research, National Cancer Institute, NIH, Bethesda, Maryland, USA; 3 Division of General Thoracic Surgery, Baylor College of Medicine, Houston, Texas, USA; 4 NIDDK Clinical Core, National Institute of Diabetes and Digestive and Kidney Diseases, NIH, Bethesda, Maryland, USA; 5 Digestive Diseases Branch, National Institute of Diabetes and Digestive and Kidney Diseases, NIH, Bethesda, Maryland, USA

**Keywords:** multiple endocrine neoplasia, neuroendocrine tumor, thymic carcinoid, thymoma, thymus

## Abstract

Patients with the multiple endocrine neoplasia type 1 (MEN1) syndrome carry germline heterozygous loss-of-function mutations in the *MEN1* gene which predisposes them to develop various endocrine and non-endocrine tumors. Over 90% of the tumors show loss of heterozygosity (LOH) at chromosome 11q13, the *MEN1* locus, due to somatic loss of the wild-type *MEN1* allele. Thymic neuroendocrine tumors (NETs) or thymic carcinoids are uncommon in MEN1 patients but are a major cause of mortality. LOH at the *MEN1* locus has not been demonstrated in thymic tumors. The goal of this study was to investigate the molecular aspects of MEN1-associated thymic tumors including LOH at the *MEN1* locus and RNA-sequencing (RNA-Seq) to identify genes associated with tumor development and potential targeted therapy. A retrospective chart review of 294 patients with *MEN1* germline mutations identified 14 patients (4.8%) with thymic tumors (12 thymic NETs and 2 thymomas). LOH at the *MEN1* locus was identified in 10 tumors including the 2 thymomas, demonstrating that somatic LOH at the *MEN1* locus is also the mechanism for thymic tumor development. Unsupervised principal component analysis and hierarchical clustering of RNA-Seq data showed that thymic NETs formed a homogenous transcriptomic group separate from thymoma and normal thymus. *KSR2* (kinase suppressor of Ras 2), that promotes Ras-mediated signaling, was abundantly expressed in thymic NETs, a potential therapeutic target. The molecular insights gained from our study about thymic tumors combined with similar data from other MEN1-associated tumors may lead to better surveillance and treatment of these rare tumors.

Patients with the multiple endocrine neoplasia type 1 (MEN1) syndrome (OMIM ID: 131100) carry germline heterozygous loss-of-function mutations in the *MEN1* gene which predisposes them to the development of various endocrine and non-endocrine tumors (Chandrasekharappa *et al.* 1997, Lemmens *et al.* 1997). Over 90% of these tumors have been shown to undergo biallelic inactivation of the *MEN1* tumor suppressor gene by somatic loss of the WT allele resulting in loss of heterozygosity (LOH) at chromosome 11q13, the *MEN1* gene locus (Thakker *et al.* 2012). An uncommon manifestation of MEN1 is thymic neuroendocrine tumor (thymic NET), also referred to as thymic carcinoid, which in MEN1 patients is a major cause of mortality (Goudet *et al.* 2010). In contrast to the other frequent NETs in MEN1 patients (pancreas, parathyroid, and pituitary), LOH at the *MEN1* locus has not been demonstrated in MEN1-associated thymic tumors (Teh *et al.* 1998, Gibril *et al.* 2003, Pan *et al.* 2005). Therefore, it is generally thought that thymic tumor development in MEN1 patients is dependent on other somatic molecular events rather than a second hit to the *MEN1* gene, and its pathogenesis is largely unknown. The lack of knowledge of its pathogenesis limits the ability to explore therapies directed at this tumor, which is the most aggressive of all MEN1-associated tumors. The goal of this study was to investigate the molecular events contributing to thymic tumor development in our well-characterized cohort of MEN1 patients by evaluating LOH at the *MEN1* locus and by utilizing transcriptomics to identify possible molecular hits that may lead to tumor development and could be considered for targeted therapy.

This study was conducted under the approval of the Institutional Review Board of the National Institutes of Health (NIH). All subjects who participated in this study provided written informed consent. We performed a retrospective chart review of 294 patients with *MEN1* germline mutations from 2 long-standing natural history protocols at our institute (NCT00001277 and NCT00001345) and identified 14 patients (4.8%) with thymic tumors (12 males and 2 females), including 6 patients previously reported (Gibril *et al.* 2003).

Histology and immunoreactivity for neuroendocrine differentiation markers (chromogranin A and synaptophysin) confirmed the neuroendocrine phenotype in 12 tumors. Two tumors were confirmed as thymoma, of which one showed neuroendocrine differentiation. Formalin-fixed paraffin-embedded (FFPE) tissue sections were obtained from thymic tumors of patients who underwent surgery at the NIH Clinical Center between 1980 and 2020. FFPE sections of normal thymus tissue were obtained from commercial sources (Zyagen, San Diego, CA, USA; US Biomax, Derwood, MD, USA) (*n* = 2), from NIH patients who underwent parathyroid surgery and removal of the normal thymus (*n* = 3), or adjacent normal of a patient with MEN1 who underwent surgery for thymic carcinoid (*n* = 1). Microdissection of the FFPE tissue sections was performed to separate the tumor tissue from the stroma. DNA and RNA were isolated using DNAstorm FFPE DNA and RNAstorm FFPE RNA extraction kits (Cell Data Sciences, Fremont, CA, USA), respectively. Genomic DNA was isolated from whole blood samples using the iPrep Purification Instrument (Thermo Scientific). LOH at the *MEN1* locus in tumor DNA was ascertained by Sanger sequencing of PCR products encompassing the region with germline *MEN1* mutation or PCR-based analysis of blood and tumor DNA with polymorphic microsatellite markers at chromosome 11q13, using previously published or newly designed primers. All primer sequences are available upon request. RNA-Sequencing (RNA-Seq) and analysis was performed by a commercially available sequencing service (QuickBiology, Pasadena, CA, USA). The trimmed mean of M values (TMM) method in edgeR package was used to normalize the gene expression, and differentially expressed genes were identified. Genes showing altered expression with edgeR multiple testing adjusted *P* value < 0.05 and more than 1.5-fold changes were considered differentially expressed.

The 13 different heterozygous *MEN1* germline mutations identified in the 14 cases with thymic tumors (patients 4 and 8 were from the same family) were scattered throughout the entire *MEN1* coding region without specific clustering (Fig. 1A and B). However, we observed a preponderance (85%) of protein-truncating mutations (frameshift and nonsense). Five of the 13 mutations were not reported previously (patients 4, 6, 9, 12, and 13).

**Figure 1:**
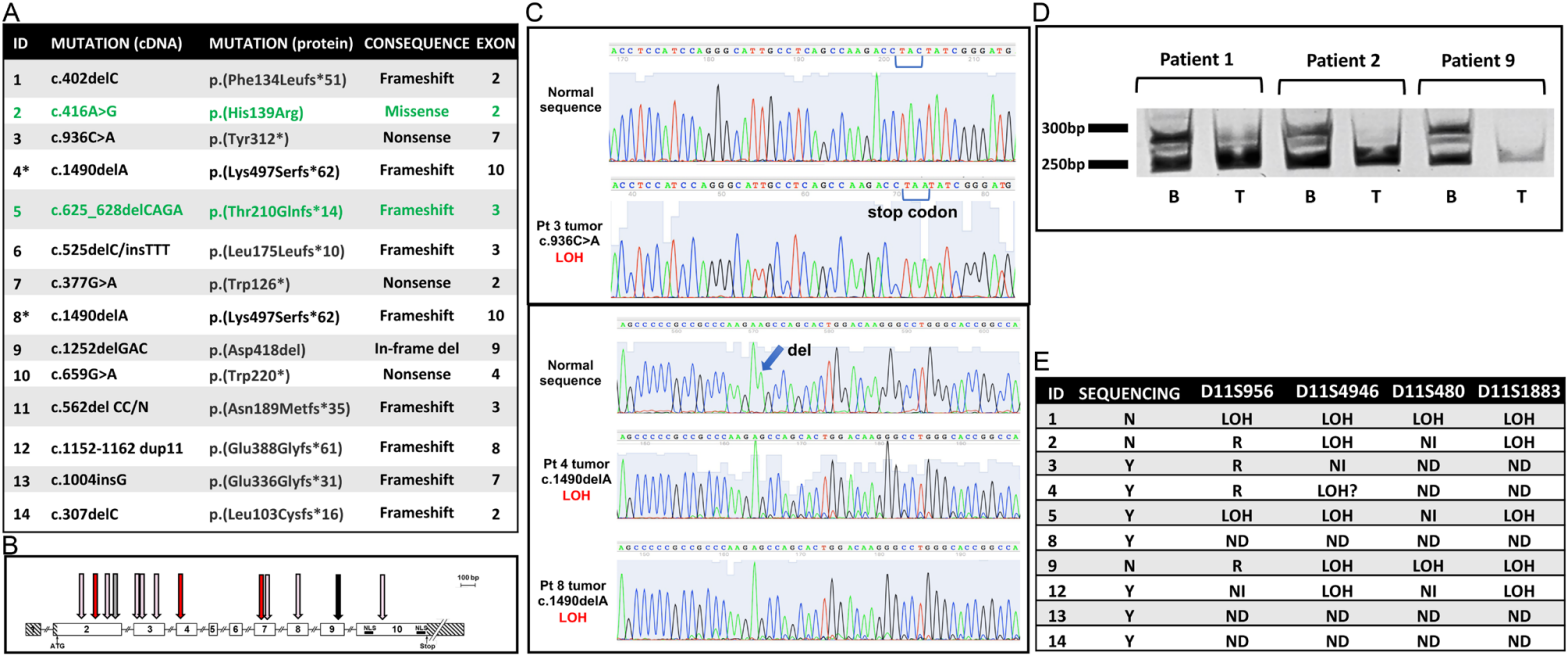
*MEN1* germline mutations and somatic loss of heterozygosity (LOH) at the *MEN1* locus in thymic tumors. (A) Germline heterozygous *MEN1* mutations in 14 MEN1 patients. Thymomas are indicated in green, and the two patients from the same family are indicated with an asterisk. Mutation nomenclature is based on reference sequence NM_130799.2 (menin isoform 2 that encodes 610 amino acids). (B) Location of 13 *MEN1* germline mutations on the diagram of the *MEN1* gene. Untranslated regions are indicated by hatched lines on either side of the gene diagram, the numbers inside the boxes indicate exon number, and NLS marks the two nuclear localization signals. The location of the mutations is indicated by the down arrows (pink for frameshift, red for nonsense, gray for missense, and black for in-frame deletion). (C) LOH detected by Sanger sequencing. DNA sequence peaks are shown for normal sequence and tumor sequence of representative samples with LOH at the germline heterozygous *MEN1* mutation. (D) LOH detected by polymorphic microsatellite marker analysis. Agarose gel electrophoresis of *MEN1* locus marker D11S1883 showing PCR products from normal DNA (B, blood) with two bands and tumor DNA (T, tumor) with one band indicating LOH in the tumor DNA. (E) Summary table showing LOH detected in 10 tumors by Sanger sequencing (Y = yes, N = no) or the indicated polymorphic microsatellite markers at the *MEN1* locus (R = retained, NI = non-informative, ND = not done). LOH in the tumor of patients 6, 7, 10, and 11 was not detected by Sanger sequencing of tumor DNA and could not be assessed with polymorphic microsatellite markers because blood DNA was not available. Previously, LOH was evaluated by polymorphic microsatellite markers in four of six tumors included in this study (patients 1, 7, 10, and 11) (Gibril *et al*. 2003). Regarding lack of LOH in the tumor of patient 1 in the previous study, it is highly likely that a different FFPE tissue block of the surgically removed tissue was used in this study.

Somatic LOH at the *MEN1* locus was identified in 10 tumors including the 2 thymomas (7 through direct Sanger sequencing and an additional 3 using polymorphic markers) (Fig. 1C,D, and E). Though thymic NETs have long been associated with MEN1, this is the first time that LOH at the *MEN1* locus has been shown in multiple thymic tumors demonstrating that, despite previous unsuccessful attempts to find LOH (Teh *et al.* 1998, Gibril *et al.* 2003, Pan *et al.* 2005), the Knudson two-hit hypothesis also applies to the thymic tumors of MEN1 syndrome, with LOH as the apparent mechanism of tumorigenesis (Knudson 1971). In addition, we showed that not only thymic NETs but also thymomas should be considered as a manifestation of the MEN1 syndrome. It is unclear why *MEN1* LOH was not detected in these tumors in previous studies. One possibility is the fact that these tumors are interspersed with normal vascular and stroma tissue due to their invasiveness. Other possible reasons that may have hindered the definitive assessment of molecular abnormalities in previous studies could be the sampling of tissue sections that lack the representative tumor part or the lack of microdissection of the tumor tissues.

Whole transcriptome analysis of MEN1-associated thymic tumors has not been reported. We performed RNA-Seq analysis of 11 thymic NETs, 2 thymomas, and 6 normal thymuses. Unsupervised principal component analysis and hierarchical clustering based on all genes expression showed that the 19 samples separated into at least 2 clusters. Thymic NETs formed a homogenous group distinct from the two thymomas which showed clustering similar to but likely distinct from the normal thymus samples (Fig. 2A and B).

**Figure 2:**
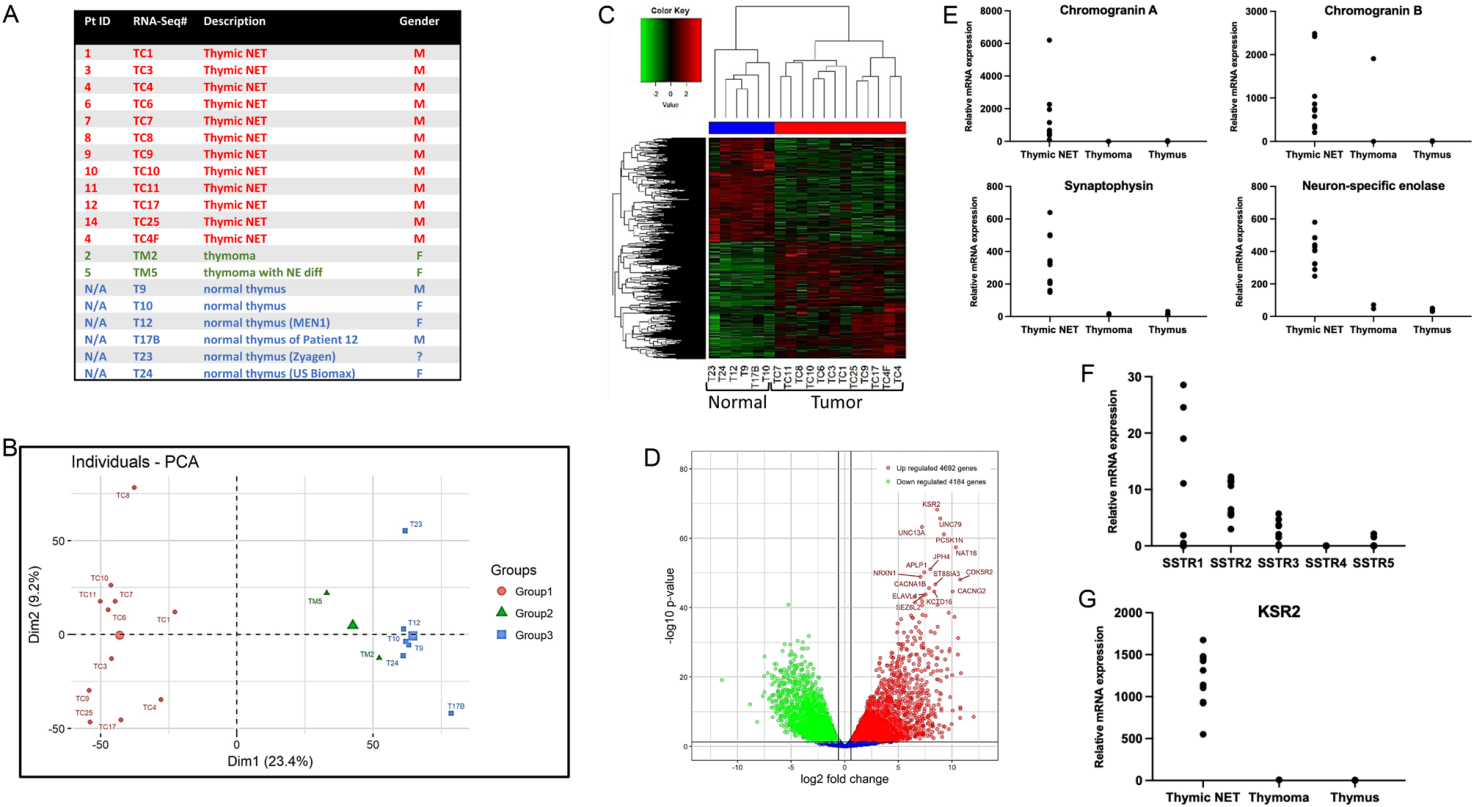
RNA-Sequencing data analysis of MEN1-associated thymic tumors compared to normal thymus samples. (A) RNA samples used for RNA-Sequencing (RNA-Seq) with corresponding patient ID. Thymic NETs (TC, thymic carcinoids) are indicated in red, thymomas (TM) in green, and normal thymuses (T) in blue. RNA was isolated from formalin-fixed paraffin-embedded (FFPE) tissue sections. TC4F represents a control RNA sample isolated from frozen tissue corresponding to FFPE tissue sample (TC4) of patient 4, to compare the RNA quality of frozen vs FFPE tissues. (B) Principal component analysis of the RNA-Seq reads of all thymic tumors and normal thymus samples. Group 1 contains thymic NETs (*n* = 11, red), group 2 contains thymomas (*n* = 2, green), and group 3 contains normal thymuses (*n* = 6, blue). The thymic NETs cluster separately from the thymomas and normal thymuses, with the thymoma clustering separately near the normal thymus. Percent variation is shown for Dim1 (dimension 1) and Dim2 (dimension 2). (C) Heatmap of hierarchical clustering of RNA-Seq reads of thymic NETs compared to normal thymuses. Differentially expressed genes are shown (rows) (edgeR adjusted *P*  < 0.05, fold change >1.5). Red shows high expression and green shows low expression. Similar gene expression data were obtained from RNA-Seq reads of frozen (TC4F) vs FFPE (TC4) tissue sample of patient 4, validating the good quality of the RNA samples isolated from FFPE tissues in this study. (D) Volcano plot of differentially expressed genes in thymic NETs compared to normal thymuses. Fold change (x-axis) and significance levels (y-axis) are shown for the differential expression of all genes. Up- and down-regulated genes are shown in red and green, respectively, and the genes with no change are shown in blue (below the line of significance). The top 15 differentially expressed genes are labeled. (E) RNA-Seq reads for the indicated genes (neuroendocrine differentiation markers) in thymic NETs, thymomas, and normal thymuses. Y-axis shows TMM-normalized transcript read counts per million. (F) RNA-Seq reads for somatostatin receptors (SSTRs) in thymic NETs. Y-axis shows TMM-normalized transcript read counts per million. (G) RNA-Seq reads for KSR2 in thymic NETs, thymomas, and normal thymuses. Y-axis shows TMM-normalized transcript read counts per million.

Of 19,657 identified transcripts from protein-coding genes, a set of 8876 genes was significantly differentially expressed between the thymic NETs and the normal thymus groups. Of these, 4692 genes were upregulated and 4184 were downregulated in thymic NETs compared to normal thymuses (Fig. 2C and D). Relative to the 6 normal thymuses and 2 thymomas, the 11 thymic NETs had significantly higher mRNA for neuroendocrine markers (more than 100-fold, *P*  < 0.02) chromogranin A, chromogranin B, neuron-specific enolase, and synaptophysin, confirming the neuroendocrine origin (Fig. 2E). Curiously, the thymoma with neuroendocrine differentiation had high chromogranin B but without significant expression of other neuroendocrine markers. Among the tumors with or without LOH at the *MEN1* locus, there was no significant difference in *MEN1* gene expression. Further analysis may help to determine whether the mutant transcript is overrepresented to compensate for the lack of normal *MEN1* transcript. Immunostaining for menin was not preformed.

NETs are known to express somatostatin receptors (SSTRs) that allow tumors to be imaged as well as treated with somatostatin analogs due to their antiproliferative effects. Given the clinical relevance of SSTRs, we evaluated the expression of the five known SSTR-encoding genes in the RNA-Seq data of our patient samples. *SSTR1, SSTR2*, and *SSTR3* were most frequently expressed across the thymic NETs cohort, while *SSTR4* was not detectable in any of the 11 samples, and *SSTR5* was only detectable in 2 of the 11 samples (Fig. 2F). SSTR-based imaging studies, OctreoScan or the more sensitive ^68^Gallium-DOTATATE PET/CT, was completed for 14 patients in this study, and imaging was positive in 91% (*n* = 11/12) and 100% (*n* = 3), respectively, consistent with SSTR expression.

Out of the 4692 significantly upregulated differentially expressed genes in thymic NETs, *KSR2* (Kinase Suppressor of Ras 2) was the most significantly upregulated (390-fold compared to normal thymuses, *P*  < 0.01) (Fig. 2D and G). The *KSR2* gene encodes for an intracellular protein that acts as a molecular scaffold for the Ras/Raf/MEK/ERK signaling pathway to promote Ras-mediated signaling. KSR2 connects Raf to its substrates MEK and ERK, and it is also involved in AMP kinase, RET, and Notch signaling (Fernandez *et al.* 2012). KSR2 has been reported to play a role in many diseases including early onset obesity, metabolic syndrome, insulin resistance, non-small cell lung cancer, melanoma, endometrial cancer, and invasive ductal carcinoma of the breast. Potential targeted therapy with available small molecule inhibitors of KSR2 has been an area of significant interest (Dhawan *et al.* 2016).

In conclusion, we show for the first time that, somatic LOH at the *MEN1* locus is the mechanism for thymic tumor development in MEN1 patients. LOH was also found in the thymomas suggesting that they are also a manifestation of the MEN1 syndrome. One limitation of our study is that not all tumor samples showed evidence of LOH at the *MEN1* locus, likely because of incomplete separation of tumor and non-tumoral cells. We found that thymic NETs formed a homogenous transcriptomic group that showed separation from thymomas and normal thymus samples. Separate clustering of tumors may indicate that thymic NETs and thymomas result from alterations in distinct pathways downstream of loss of the *MEN1*-encoded protein menin. Another limitation of our study is that we compared the transcriptome of thymic tumors to normal thymuses, given that no cell-of-origin has been shown for MEN1-associated thymic tumors. Further studies comparing our valuable dataset of thymic tumor RNA-Seq with the cell-of-origin will be informative to reveal gene signatures specific to thymic tumors and the precise molecular mechanisms of thymic tumor pathogenesis. The molecular insights from our study coupled with an understanding of the modifying factors that result in the unique and aggressive behavior of thymic NETs compared to the behavior of the other NETs in MEN1 patients may lead to better surveillance and treatment for patients with thymic tumors, which is very limited at present.

## Declaration of interest

J E B is a full-time employee of AstraZeneca. The other authors have nothing to disclose.

## Funding

This work was supported by the Intramural Research Program of the NIH: National Institute of Diabetes and Digestive and Kidney Diseases (grant number 1ZIADK075085-08) and National Cancer Institute (grant number 1ZIABC011115-13).

## Data access

RNA-Seq files of raw data and processed data have been submitted to GEO under accession number GSE177522.

## Author contribution statement

Study design (A M, J E B, S K A), clinical samples and associated data (A M, D S S, G K, J D R, J E B, J M W, L S W, M F W, R T J, R T R, S J, W F S), data procurement and data analysis (A M, J E B, S K A), manuscript writing (A M, J E B, L S W, S K A), review and editing manuscript (A M, D S S, G K, J D R, J E B, J M W, L S W, M F W, S J, R T J, R T R, S K A, V I P, W F S).
